# Thoroughness in Surgery

**Published:** 1899-12-23

**Authors:** 


### Thoroughness in Surgery.

When called upon to sing the pæans of modern surgery, it is not unnatural that men should seek for text and for example in those fields which have been explored only within the time of the present generation. But to those who have watched the evolution of modern methods, and have observed the steps by which some of the more ordinary operations which are commonly performed to-day have arrived at their present position, it has become obvious that the change which has taken place in surgery during the last quarter of a century is widespread and deep-seated, and that besides the introduction of new methods and new operations, and the intrusion of the surgeon's knife into new regions where before it was held to have no place, there has crept over the whole face of surgery, whether in the opening of a boil or in the excision of a tumour, a new character which can, perhaps, be best expressed by the word thoroughness. To those outside the profession who merely hear of what doctors are doing, and have but a general idea of what is going on in hospitals, the striking fact in surgical progress is the enormous number of operations that are being done; but to those inside the striking fact is the enormous amount of care which is being taken over each operation, and this not merely in the doing of it, but in the planning of the procedure, and especially in so ordering it that it shall thoroughly effect its purpose. Indeed, if we were asked, What is the main characteristic of modern surgery? much as we might feel tempted to reply that it is boldness, or accuracy, or cleanliness, asepticism, or what not, we should probably in the end come round to the assertion that its great characteristic, as displayed in the principal operating theatres, is its thoroughness. A good example of the sort of thoroughness to which we refer is to be found in the description given by Mr. Watson Cheyne, at the last meeting of the Medical Society, of the modern method of dealing with tuberculous glands in the neck. In regard to the excision of scrofulous cervical glands it is worth noting, as showing how quickly changes occur, that it was only in 1885 that Mr. Pridgin Teale showed how necessary it was, in removing such glands, to trace up the chain, burrowing even under the deep fascia, so as to get to the root of the mischief. This was a great advance, for, as Mr. Teale says, this mode of dealing with such glands was at that time scarcely known in surgery. Since then, however, an enormous amount of gland surgery has been done, and often with the happiest results. Not always, however. In the early days, especially when the method adopted was to "grub out" the glands, the results were variable, and by no means always successful, and those who have watched what has been done by various surgeons in this sort of work can hardly doubt that the growing certainty and satisfaction with which gland surgery has been rewarded during recent years has been largely associated with a growing thoroughness in the operations which have been undertaken.

Passing over then the eras of simple incision, of drainage, and of scraping, and the interludes of aspiration, injection with iodoform and other methods, we come to that of excision, and when it is asked how shall excision be done, modern surgery, speaking in the person of the Harveian Lecturer, says it must be done thoroughly. "In operations on tuberculous glands one must not be content with shelling out the infected glands, but must remove the whole of the glandular area, whether the glands are visibly affected or not." What, then, is the operation? Taking the most common position, namely, the upper part of the anterior triangle of the neck, one must remove not only the whole of the fat and fascia present in the anterior triangle, leaving the vessels, nerves, and other structures thoroughly clean, but if one is to avoid recurrence one must also thoroughly clean out all the tissues underneath the sterno-mastoid, so that the muscles on which the sterno-mastoid rests are completely bared. It may quite well happen that the jugular vein may have to be divided above and below and removed along with the glands. In any case it must be exposed. Other large veins—the superior thyroid, the lingual, and the facial—will have also to be dealt with, the carotid artery and the vagus nerve will have to be separated from the jugular for the removal of the latter, the spinal accessory nerve must be isolated and protected, the branch of the spinal accessory to the trapezius must be watched for and preserved, as also must the descendens noni. This is a thorough operation, and is typical of what is being done in many fields.

We need hardly say that such operations as these have a social as well as a surgical aspect. No one can pretend that highly elaborate surgery of this kind can be rendered accessible to the lower middle classes except in hospitals. Yet there they can in too many instances only obtain it by entering these institutions under pretences which are more or less false. This ought to be rectified. Patients should pay what they can. Even when they have paid their utmost they will have received a charity for which they can never be too thankful.

